# Rheology of Concentrated Polymer/Ionic Liquid Solutions: An Anomalous Plasticizing Effect and a Universality in Nonlinear Shear Rheology

**DOI:** 10.3390/polym11050877

**Published:** 2019-05-14

**Authors:** Zhonghua Liu, Wei Wang, Florian J. Stadler, Zhi-Chao Yan

**Affiliations:** College of Materials Science and Engineering, Shenzhen Key Laboratory of Polymer Science and Technology, Guangdong Research Center for Interfacial Engineering of Functional Materials, Nanshan District Key Lab for Biopolymers and Safety Evaluation, Shenzhen University, Shenzhen 518055, China; liuzhonghua2016@email.szu.edu.cn (Z.L.); wangsoftmatter@gmail.com (W.W.); fjstadler@szu.edu.cn (F.J.S.)

**Keywords:** polymer/ionic liquid solution, plasticizing effect, linear and nonlinear rheology

## Abstract

An anomalous plasticizing effect was observed in polymer/ionic liquid (IL) solutions by applying broad range of rheological techniques. Poly(ethylene oxide)(PEO)/IL solutions exhibit stronger dynamic temperature dependence than pure PEO, which is in conflict with the knowledge that lower-*T*_g_ solvent increases the fractional free volume. For poly(methy methacrylate)(PMMA)/IL solutions, the subtle anomaly was detected from the fact that the effective glass transition temperature *T*_g,eff_ of PMMA in IL is higher than the prediction of the self-concentration model, while in conventional polymer solutions, *T*_g,eff_ follows the original Fox equation. Observations in both solutions reveal retarded segmental dynamics, consistent with a recent simulation result (*Macromolecules*, 2018, 51, 5336) that polymer chains wrap the IL cations by hydrogen bonding interactions and the segmental unwrapping delays their relaxation. Start-up shear and nonlinear stress relaxation tests of polymer/IL solutions follow a universal nonlinear rheological behavior as polymer melts and solutions, indicating that the segment-cation interaction is not strong enough to influence the nonlinear chain orientation and stretch. The present work may arouse the further theoretical, experimental, and simulation interests in interpreting the effect of complex polymer-IL interaction on the dynamics of polymer/IL solutions.

## 1. Introduction

Polymer/ionic liquid (IL) mixtures have received great attention as novel materials with versatile applications, such as plastic electronics [1,2], gas separation membranes [3,4,5,6,7,8], luminescent and electrochromic devices [9,10,11], high-capacitance gate dielectric [12,13], fuel cells [14], and solutions for cellulose spinning [15]. In mixtures, ionic liquids provide conductive and gas separating properties, while polymers act as skeletons to immobilize ILs [1]. To develop robust materials maintaining the functionality of pure IL, a thorough understanding of static and dynamic behaviors of polymers in ILs is required.

The typical neutral polymers which are miscible with ILs are poly(ethylene oxide)(PEO) [16,17,18,19,20,21,22] and poly(methy methacrylate)(PMMA) [23]. For PEO/IL mixtures, small-angle neutron scattering measurements were performed in a variety of ILs such as 1-butyl-3-methylimidazolium tetrafluoroborate ([Bmim][BF_4_]) [17,18], ethylammonium nitrate (EAN) [20,21], lithium tetraglyme bis(trifluoromethanesulfonyl)amide ([Li(G4)][Tf_2_N]) [22], and propylammonium nitrate (PAN) [21]. [Bmim][BF_4_] is found to be a moderately good solvent for PEO, showing an excluded volume exponent of 0.55 at 80 °C [17], while EAN and [Li(G4)][Tf_2_N] are better solvents considering the more expanded coil in them [20,21,22]. The solvent quality of PAN, however, is close to θ condition, worse than EAN which has the shorter alkyl group [21]. In addition, the chain expansion of PEO of the same molecular weight was found to be more modest in ILs than in good solvents such as water [17,21]. Liu et al. [16] performed rheological measurement on PEO in different imidazolium ILs. The molecular weight dependence of intrinsic viscosity reveals that the solvent properties of ILs become better with increasing anion size, but with unknown reason, the concentration scaling exponents of specific viscosity were determined to be 1.0, 2.0, and 4.7 in dilute, semidilute unentangled, and semidilute entangled regimes respectively, independent of the kind of IL. On the computational side, Yethiraj et al. [24,25] reported that PEO in [Bmim][BF_4_] adopts an extended conformation (excluded volume exponent *v* ≈ 0.9) at room temperature and a random conformation (*v* ≈ 0.5) around 400 K. Recently, Son et al. [19] recovered the experimental exponent (*v* ≈ 0.56) by simulation in the temperature range of 300–600 K, confirming the conformation of PEO in [Bmim][BF_4_] is in between a self-avoiding walk and an ideal chain. Costa et al. [26] found the cation dependence that PEO adopts more extended conformation in 1-butyl-3-methylimidazolium hexafluorophosphate ([Bmim][PF_6_]) than in 1,3-dimethylimidazolium hexafluorophosphate ([Dmim][PF_6_]). As for PMMA/IL mixtures, the research is limited, as only one systematically experimental study of concentrated PMMA/1-ethyl-3-methylimidazolium bis(trifluoromethanesulfonyl)imide ([Emim][Tf_2_N]) solutions by calorimetric and rheological techniques was reported to the best of the authors’ knowledge [23]. In these solutions, PMMA and [Emim][Tf_2_N] are homogenously mixed in the whole concentration range. [Emim][Tf_2_N] acts as plasticizer to reduce the glass transition temperature (*T*_g_), the plateau modulus, and the terminal relaxation time of PMMA.

**Scheme 1:**
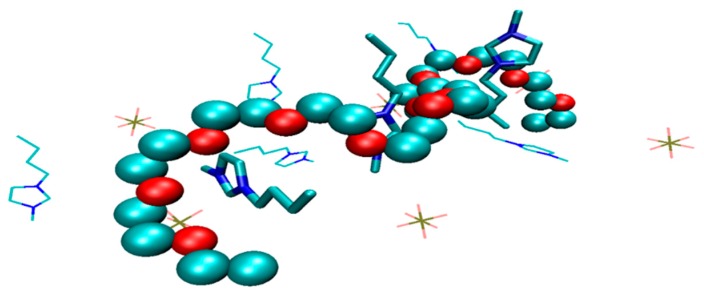
Representative illustration showing that chain segments (PEO) wrap around the cations of ionic liquids ([Bmim][PF_6_]) due to hydrogen bonding interaction.

Tsuda et al. [27] and Lee et al. [28] performed NMR measurements on PEO (and derivatives) in imidazolium ILs, revealing hydrogen bonding interaction between H atoms on the C2 position of the imidazolium ring and the ether oxygen on PEO. This interaction benefits the solvation of PEO and governs the phase separation mechanism. Asai et al. [29] and Xiao et al. [30] further confirmed this hydrogen-bonding interaction by high-energy X-ray diffraction and Fourier-transform infrared spectroscopy, respectively. Recently, a simulation based on ab initio force field [19] revealed that the cations of [Bmim][BF_4_] are prone to be coordinated by oxygens on polymer segments due to the hydrogen bonding interaction (Scheme 1). Such a coordination effect divides the polymer conformation into ring-like and linear segments, with the ring-like portion wrapping around cations. Slow dynamics arise from the unwrapping of chain, as the mean-square displacement shows a confinement regime (~*t*^1/4^), which corresponds to the time scale for the dissociation and exchange of the coordinating ions to segments.

Despite the progress in simulation, the influence of wrapping effect on dynamics has not been experimentally detected so far. Two candidate techniques are quasielastic neutron scattering (QENS) and NMR. However, these two methods require deuterated IL and deuterated (or ^13^C-labeled) polymer, respectively. Samples are less available, and the deuterations might influence the polymer-IL interactions. Alternatively, rheology is an accessible technique without component labeling. To investigate such subtle dynamic behavior, we focus on the concentrated regime, in which polymer response dominates the rheological data. By analyzing the dynamic temperature dependence, the parameters, including the fractional free volume, the activation energy, and the effective glass transition temperature, can be extracted, which could quantitatively measure the influence of IL molecules on the segmental dynamics.

Another question underlying the polymer/IL solutions is whether the interaction could influence the nonlinear rheological behavior. The current research on extensional and shear flow of conventional polymer solutions focuses on the flow-induced friction reduction and nematic interaction [31,32,33,34,35]. However, these conventional solutions do not have specific interactions as complex as observed in polymer/IL systems. The nonlinear rheology of polymer/IL solutions could reveal the role of the wrapping effect on chain deformation under flow or large strain. In addition, such study bears an application importance to guide the processing of polymer-IL solid electrolytes and gel spinning.

**Scheme 2:**
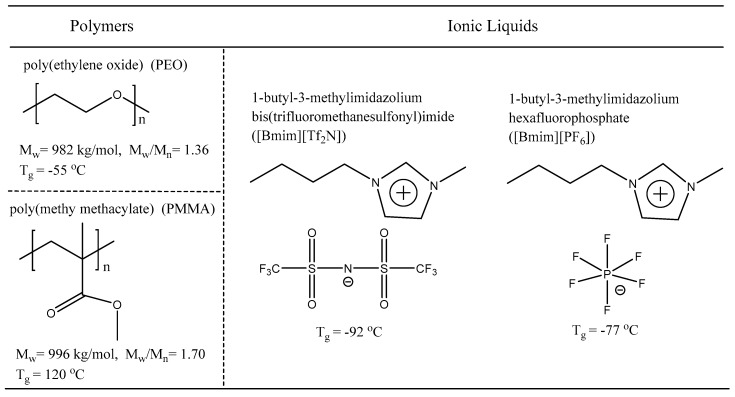
Structure, molecular weight, and *T*_g_ of PEO, PMMA, [Bmim][PF_6_], and [Bmim][Tf_2_N].

In this work, we employed entangled PEO/[Bmim][PF_6_], PEO/[Bmim][Tf_2_N], and PMMA/[Bmim][Tf_2_N] as model systems (Scheme 2). [Bmim][PF_6_] and [Bmim][Tf_2_N] are θ and good solvents for PEO [16], with their differences in *T*_g_ from PEO (Δ*T*_g_) being 22 and 37 °C, respectively. Such small Δ*T*_g_ makes these two ILs quasi-iso-*T*_g_ solvents for PEO. The subtle interaction-induced change on segmental dynamics can be sensed by comparing the temperature dependence of horizontal shift factor *a*_T_ (or fractional free volume) to that of polymer melt [36]. On the other hand, Δ*T*_g_ in PMMA/[Bmim][Tf_2_N] is as large as 212 °C. The strong plasticizing effect makes it difficult to directly detect the subtle interaction effect through *a*_T_. In this case, we resort to the concept of effective glass transition temperature *T*_g,eff_, a measure of segmental dynamics in mixtures. By analyzing the temperature dependence of normalized terminal relaxation time, we can extract *T*_g,eff_ of PMMA in each solution. According to the comparison of *T*_g,eff_ among IL solutions, conventional solutions, and theoretical predictions, the contribution of polymer-IL interactions can be evaluated. In nonlinear rheology, start-up shear and nonlinear stress relaxation measurements were performed to investigate the possible effect of interaction on chain deformation under shear flow and large strain. Finally, concluding remarks are summarized.

## 2. Experimental Section

### 2.1. Sample Information

Poly(ethylene oxide) (PEO) and poly(methy methacylate) (PMMA) were purchased from Sigma-Aldrich. The weight-average molecular weight (*M*_w_) and distribution (*M*_w_/*M*_n_) were characterized in previous report [16,36], to be *M*_w_ = 982 kg/mol and *M*_w_/*M*_n_ = 1.36 for PEO [16], and *M*_w_ = 996 kg/mol and *M*_w_/*M*_n_ = 1.7 for PMMA [36]. Ionic liquids, 1-butyl-3-methylimidazolium bis(trifluoromethanesulfonyl)imide ([Bmim][Tf_2_N]) and 1-butyl-3-methylimidazolium hexafluorophosphate ([Bmim][PF_6_]), were purchased from the Center for Green Chemistry and Catalysis of Lanzhou Institute of Chemical Physics, Chinese Academy of Sciences. The residual water in ILs was removed by rotary evaporation at 80 °C, followed by vacuum drying for 48 h at 80 °C with phosphorus pentoxide (P_2_O_5_) until water content is undetectable by Karl Fischer titration. The glass transition temperatures of polymers and ILs were measured by differential scanning calorimetry (DSC) (Hitachi DSC 7020) at a 10 °C/min heating rate. The DSC traces are shown in Figures S1 and S2 in Supporting Information (SI). For [Bmim][Tf_2_N] and PEO, a quenching procedure was used before heating in order to minimize the crystallization. The glass transition temperature was identified as the peak of the derivative DSC heat flow curve. Sample structures and properties are shown in Scheme 2.

### 2.2. Preparation of Polymer/IL Solutions

PEO and ILs were mixed and heated to approximately 80 °C with stirring until complete dissolution. The homogenized solutions were then dried under vacuum with P_2_O_5_ for 24 h to remove moisture. All solutions were prepared on a weight basis. The polymer volume fraction *ϕ* was calculated based on reported density [37,38] and the assumption of no volume change on dissolution. PMMA/Bmim[Tf_2_N] was dissolved with the aid of cosolvent methylene chloride. After complete evaporation of cosolvent in a fume hood, solutions were further dried under vacuum at 80 °C for 24 h to remove the residual cosolvent and moisture.

The concentrations of PEO/IL solutions are limited to 0.10 and 0.055 due to the crystallization of PEO at high concentrations [16], while the amorphous PMMA can mix with ILs in the whole concentration range [23]. The miscibility of ILs with polymers can be judged according to Kamlet-Taft parameters (25 °C). For [Bmim][PF_6_] and [Bmim][Tf_2_N], the hydrogen bond acidity *α* of cations is respectively 0.54 and 0.55, while the hydrogen bond basicity *β* of anions is respectively 0.42 and 0.44 [39], indicating their similar ability to donate and accept hydrogen bond. The polarizability of π^*^ is 0.90 for [Bmim][PF_6_] and 0.83 for [Bmim][Tf_2_N] [39]. Both values are relatively lower compared with other ILs, which are mostly above 1.0 [39]. Such low polarizability is believed to favor the solubility of neutral polymers like PEO [16] and PMMA [23], since less energy is required to decouple the ion pairs [40]. [Bmim][Tf_2_N] has a lower polarizability than [Bmim][PF_6_], so [Bmim][Tf_2_N] can dissolve both PEO and PMMA, while [Bmim][PF_6_] can only dissolve PEO. Furthermore, the hydrogen bond donation from cations could also contribute to the dissolution of neutral polymers through interaction with oxygen on polymer chains [19,27,28,29].

### 2.3. Rheological Measurement

Linear rheological measurement was conducted based on dynamic frequency sweeps, while nonlinear rheological measurements include both start-up shear test, which measures the transient viscosity upon applying a shear rate, and the step-strain test, which measures the stress relaxation after imposing a step strain deformation. Linear and nonlinear rheology of PEO/IL solutions was measured using a cone-plate geometry with cone angle of 1° and diameter of 15 mm on an Anton Paar 302 rheometer. Temperatures were controlled by Peltier plate. A hood was equipped and purged with nitrogen for protection from moisture and oxygen. For PMMA/IL, which has larger elasticity, an Anton Paar 702 rheometer equipped with cone partitioned-plate geometry (CPP) was used to minimize edge fracture. The upper plate (inner partition) has a diameter of 8 mm, surrounded by a hollow cylinder (outer partition), while the lower cone plate has a diameter of 25 mm and a cone angle of 4°. The temperature was controlled by the convective nitrogen flow in the oven.

## 3. Linear Viscoelasticity

### 3.1. PEO/IL Solutions

**Figure 1:**
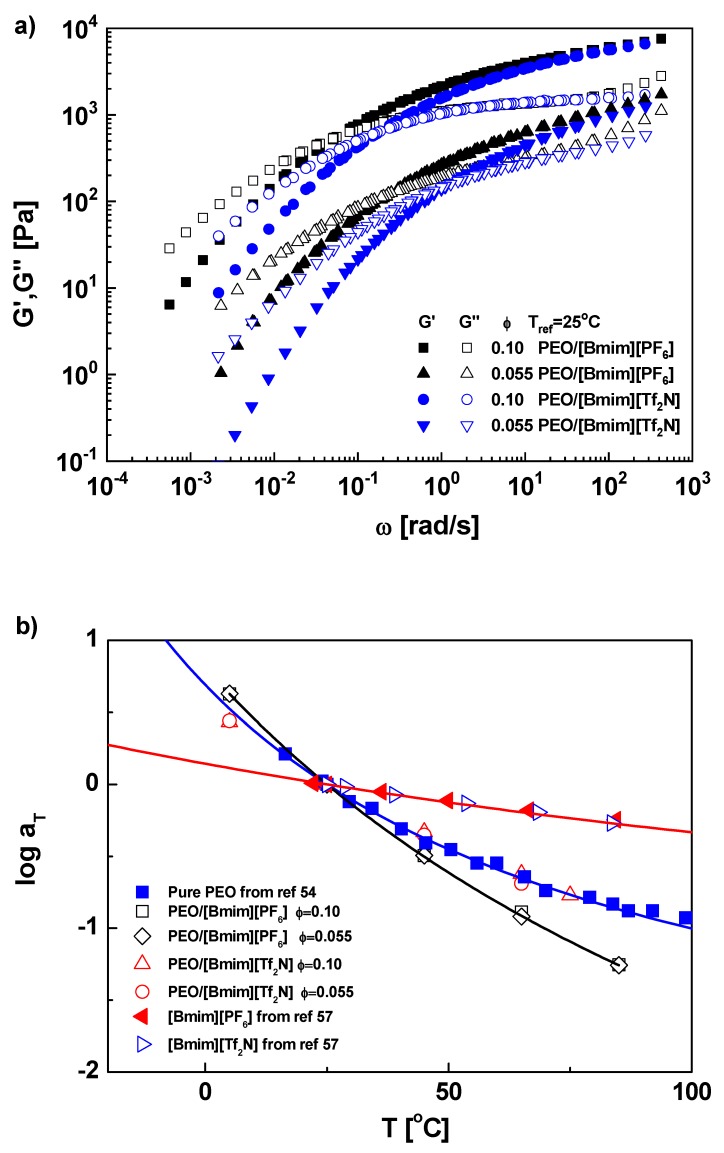

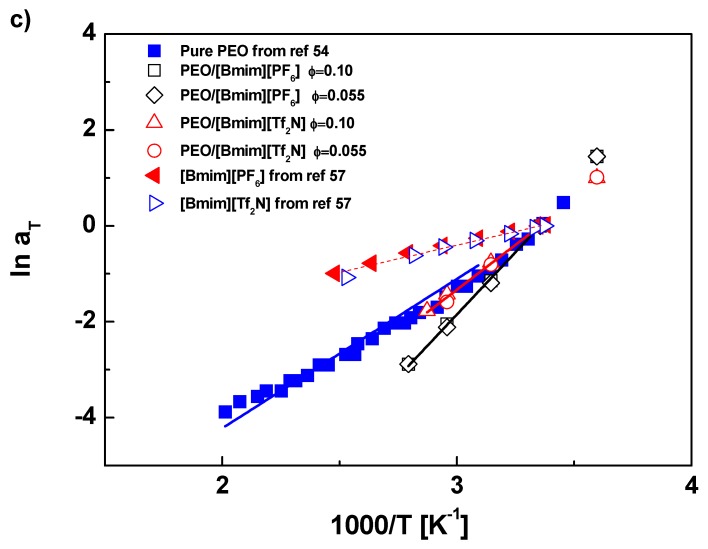
(**a**) Master curves of PEO in [Bmim][PF_6_] and [Bmim][Tf_2_N] at *T*_ref_ = 25 °C. (**b**) Horizontal shift factors *a*_T_ as function of temperature *T* with WLF fitting. (**c**) *a*_T_ as function of 1000/*T* with Arrhenius fitting, where the lower temperature limit for fitting is 45, 25 and 25 °C for PEO, PEO/[Bmim][PF_6_], and PEO/[Bmim][Tf_2_N], respectively.

The master curves of PEO solutions are shown in Figure 1a. Both horizontal and vertical shifts were performed, with vertical shift based on the density compensation method [41], where the density of ILs at different temperatures was taken from ref [42]. Two *ϕ* = 0.10 solutions (in [Bmim][PF_6_] and [Bmim][Tf_2_N]) exhibit a plateau modulus *G*_N_^0^ of 5.0 × 10^3^ Pa, with *G*_N_^0^ being defined as *G*′ at the minimum of tanδ, while for two *ϕ* = 0.055 solutions, *G*_N_^0^ is around 1.0 × 10^3^ Pa. Along with *G*_N_^0^ of pure PEO, which is reported as 1.8 × 10^6^ Pa [43], *G*_N_^0^ scales with *ϕ* by *G*_N_^0^ ~ *ϕ*^2.5^. This scaling exponent (2.5 ± 0.2, considering the uncertainty from limited data points and fitting) is on the upper limit of the reported values for conventional polymer solutions (2.0~2.5) [44], and similar with the exponent (≈2.6) in PMMA/[Emim][Tf_2_N] solutions when *ϕ* is lower than 0.2 [23]. Liu et al. [16] reported that [Bmim][Tf_2_N] and [Bmim][PF_6_] are respectively athermal and θ solvents for PEO. According to the Colby-Rubinstein theory based on tube model [45,46], PEO in both solvents should show an identical dilution exponent of 2.3 for *G*_N_^0^. The larger scaling exponent from our experiments implies the loss of entanglements. 

The entanglement number *Z* of PEO solutions is approximately estimated by *M*/(*M*_e_/*ϕ*^α^), with *M*_e_ being the entanglement molecular weight and *α* being the dynamic dilution exponent [46]. *M*_e_ is 1.6 kg/mol for PEO [43]. The value of *α* is still on debate ranging from 1.0 to 1.3 [47,48]. Here, we use *α* = 1 as previously reported in branched polymers (close to solutions) for a consistent comparison [49,50,51,52]. The estimated entanglement number *Z*_est_ = 61.3 for *ϕ* = 0.10 and *Z*_est_ = 33.7 for *ϕ* = 0.055. For comparison, we also calculate the experimental entanglement number *Z*_exp_ from *G*_N_^0^ by *M*/(*ϕρRT/G*_N_^0^) [47], which results in *Z*_exp_ = 19.4 for *ϕ* = 0.10 and *Z*_exp_ = 7.9 for *ϕ* = 0.055. *Z*_exp_ is smaller than the corresponding *Z*_est_. Such deviation may stem from the local heterogeneity in IL solutions, since local structures are probably formed when partial PEO chains wrap around cations through hydrogen bonding interaction (Scheme 1) [19]. Recently, Xiao et al. [30] observed nano-scale cluster-like heterogeneous structure in PEO/[Emim][BF_4_] solutions. Such clusters may introduce defects into entanglement network and reduce *G*_N_^0^.

PEO/[Bmim][Tf_2_N] relaxes faster than PEO/[Bmim][PF_6_], because [Bmim][Tf_2_N] has lower *T*_g_ and hence plasticize polymer more significantly than [Bmim][PF_6_]. The plasticizing effect is usually evaluated from the change of terminal relaxation time *τ*_d_. However, the rheological measurement of semi-crystalline high-*M*_w_ PEO can only be performed after complete melting of polymers, which restricts the terminal relaxation time from reaching lower temperatures. Therefore, the fitting parameters of pure PEO cannot be reliably employed to extract dynamic results (e.g., *T*_g,eff_) in solutions due to their different temperature ranges. Besides, the terminal regime can only be obtained at very high temperature, e.g., 195 °C for PEO with *M*_w_ = 932 kg/mol [53]. At such high temperatures, polymers risk thermal instability even under protection of nitrogen. For above reasons, we alternatively selected the reported data of pure PEO from NMR and dielectric measurements [54], which could reach relatively lower temperatures. The evaluation is performed by directly fitting the temperature dependence of horizontal shift factor *a*_T_. 

At *T*_g_ < *T* < *T*_g_ + 100 °C, temperature dependence of *a*_T_ could be fitted by the William-Landel-Ferry (WLF) equation [55]

(1) $$\log\left(a_{T}\right)=\frac{-C_{1}\left(T-T_{\mathrm{ref}}\right)}{C_{2}+T-T_{\mathrm{ref}}}$$

with *C*_1_ and *C*_2_ being WLF parameters at reference temperature *T*_ref_. The fractional free volume *f*/*B* at *T*_ref_ can be obtained from *C*_1_ [36,50,52]

(2) $$f/B=\frac{1}{2.303C_{1}}$$

where *B* is a constant of order unity. The value of *f*/*B* is a measure of segmental dyanmics, since it correlates with monomeric frictional coefficient *ζ*_0_ by *ζ*_0_ ≈ exp(*B/f*) [41,55]. On the other hand, at sufficiently high temperatures (typically above *T*_g_ + 100 °C), temperature dependence is usually described by the Arrhenius equation [46,47]

(3) $$a_{T}=\exp\left[\frac{E_{a}}{R}\left(\frac{1}{T}-\frac{1}{T_{\mathrm{ref}}}\right)\right]$$

with *E*_a_ being the flow activation energy and *R* being the gas constant. *E*_a_ is a measure of segmental dynamics at a given high temperature range, in positive correlation to the monomeric friction.

The critical temperature *T*_g_ + 100 °C is 45, 25, and 10 °C for pure PEO, PEO/[Bmim][PF_6_] solutions (*ϕ* = 0.10 and 0.055), and PEO/[Bmim][Tf_2_N] (*ϕ* = 0.10 and 0.055) solutions, respectively, with *T*_g_ in solutions being calculated by the Fox equation based on glass transition temperatures of pure polymer and solvent [56]. Therefore, the experimental temperature range (5~85 °C) in our study is at the crossover regime from WLF to Arrhenius fitting. For this reason, both WLF and Arrhenius fittings are performed in the following.

The horizontal shift factors of pure PEO and PEO/IL solutions are plotted in Figure 1b at reference temperature *T*_ref_ = 25 °C. The *a*_T_ data of pure PEO were from dielectric and NMR measurements [54] and of the same *T*_g_ as ours, while the *a*_T_ data of pure ILs ([Bmim][PF_6_] and [Bmim][Tf_2_N]) were from ref [57]. The WLF fitting parameters at 25 °C are *C*_1_ = 2.59 and *C*_2_ = 119 °C for pure PEO, and *C*_1_ = 1.93 and *C*_2_ = 361 °C for both [Bmim][PF_6_] and [Bmim][Tf_2_N], whose data overlap each other. The corresponding fractional free volume is thus calculated as *f/B* = 0.167 and 0.225 for pure PEO and ILs, respectively. For PEO/[Bmim][PF_6_], the *a*_T_ data at *ϕ* = 0.10 and 0.055 overlap within experimental error, indicating the change of monomeric friction (or fractional free volume) levels off when the majority in mixture is the solvent, consistent with the phenomenon in conventional polymer solutions [36,58]. Therefore, we use a single set of WLF parameters to fit PEO/[Bmim][PF_6_] solutions at both concentrations here. Surprisingly, PEO/[Bmim][PF_6_] exhibits a stronger temperature dependence of *a*_T_ than pure PEO and has WLF parameters *C*_1_ = 5.05 and *C*_2_ = 181 °C and fractional free volume *f/B* = 0.086. This result conflicts with the traditional knowledge that solvents with higher fractional free volume (*f/B* = 0.225) could plasticizes polymer with lower values (*f/B* = 0.167) [36,41,58]. One may argue that the difference of *T*_g_ between polymer and solvent is relatively small, i.e., Δ*T*_g_ ≈ 22 °C. However, for 1,4-polybutadiene (PBD)/phenyloctane (PHO), which is a conventional polymer solution with Δ*T*_g_ ≈ 24 °C, the plasticizing effect is significant [36,58]. At 25 °C, the fractional free volume of PBD increases from 0.143 to 0.211 when the polymer concentration decreases from 1 to 0.15 [36]. This comparison implies some specific polymer-solvent interactions in PEO/IL solutions which retard the segmental dynamics. 

For PEO/[Bmim][Tf_2_N], *a*_T_’s at two concentrations overlap and approximately collapse on the WLF curve of pure PEO, which means the apparent fractional free volume of PEO is not effectively influenced by [Bmim][Tf_2_N] even though Δ*T*_g_ is as high as 37 °C. Such iso-free-volume phenomenon was only reported in solutions with polymer and solvent having the same *T*_g_ or fragility [36,58], but not in lower-*T*_g_ solvents. This result suggests that the interaction-induced slow dynamics could compensate the plasticization from IL, consistent with the anomaly in PEO/[Bmim][PF_6_]. The relatively weaker temperature dependence of *a*_T_ for PEO/[Bmim][Tf_2_N] solutions is due to its smaller *T*_g_, and hence stronger plasticizing effect, with respect to [Bmim][PF_6_].

The Arrhenius fitting is shown in Figure 1c. The selected fitting range is beyond *T*_g_ + 100 °C. As the case in WLF fitting, the data at two concentrations (0.1 and 0.05) for the same kind of solution overlap, and thereby they are fitted with the same Arrhenius parameters. Good fitting results are obtained for all samples. The slight deviation in pure PEO could be attributed to the uncertainty of the crossover temperature, which may be higher than *T*_g_ + 100 °C. As a fitting parameter, the activation energy *E*_a_ is 25.9, 9.45, 43.2, and 31.1 kJ/mol for pure PEO, pure ILs ([Bmim][PF_6_] and [Bmim][Tf_2_N]), PEO/[Bmim][PF_6_], and PEO/[Bmim][Tf_2_N], respectively. Beyond expectation, the *E*_a_ value of PEO/[Bmim][PF_6_] is not in between PEO and [Bmim][PF_6_], but higher than pure PEO, indicating a more difficult segmental motion, which is consistent with the conclusion from the WLF fitting. The data of PEO/[Bmim][Tf_2_N] collapse on the PEO curve again. However, due to the different fitting ranges, PEO/[Bmim][Tf_2_N] has a larger *E*_a_ than pure PEO, implying the segmental motion in [Bmim][Tf_2_N] solutions is also slightly retarded.

At last, the success of time-temperature superposition implies the effect of polymer-IL coordination on chains’ global relaxation is insensitive to temperature, at least in this narrow experimental range. Otherwise, the superposition may fail. Actually, the wrapping effect on segmental dynamics has already been reflected in the anomalous temperature dependence of *a*_T_, which does not influence the validity of superposition.

In solid polymer electrolytes, salts are dissolved in macromolecules according to ion-coordination [59]. Such ion-coordination can act as intrachain or interchain transient crosslinker, retarding the segmental relaxation of polymers and affecting the ion transport [60,61,62,63,64,65,66]. For example, in typical PEO-salt mixtures, cations are coordinated by 5−7 ether oxygen atoms, and the cation-ether oxygen complex could reduce the mobility of monomers [67,68,69,70]. Rheological behavior of PEO is accordingly tuned by the content of salts. Harun et al. [71] studied the calorimetry and rheology of PEO-lithium salt mixtures. They found *T*_g_ increases with salt content, and the terminal relaxation is extended and broadened. Goldansaz et al. [72] investigated the rheology of PEO-nickel salt mixtures. Ether oxygen and nickel can form strong coordination, which reversibly crosslinks polymer chains and results in a secondary plateau beyond terminal regime of pure polymers. Ionic liquids are also salts, so their coordination with polymer is nonnegligible. As found in simulation [19], cations on [Bmim][BF_4_] are wrapped by ring-like PEO segments, similar with the crown-ether-like coordination of lithium cations by monomers of PEO. The time scale for dissociation and exchange of the coordinating ions to the monomers is as large as tens of nanoseconds at room temperatures. Such a survival time of confinement is comparable with the segmental relaxation time and hence provides a rigid frame to overall polymer structure [19]. Also affected by the structural heterogeneity and high viscosity of ILs, diffusions of polymers could feel confinement at short time scale, so the segmental dynamics is effectively influenced by the coordination exchange. As a result, slow dynamics arises from unwrapping the coordination, which retards the overall segmental dynamics of PEO. Although the concentration in this study is relatively high, the polymer-IL interaction still exists. Such coordination-induced slow segmental dynamics compete with the plasticizing effect from solvents. In PEO/[Bmim][PF_6_], the slow dynamics overwhelms the plasticization, so the apparent fractional free volume is anomalously small. In PEO/[Bmim][Tf_2_N] with solvent having lower *T*_g_, the slow dynamics and the plasticization reach a balance, so the apparent fractional free volume keeps unchanged.

### 3.2. PMMA/IL Solutions

To check the universality of this anomalous plasticizing effect, a solution with large Δ*T*_g_, PMMA/[Bmim][Tf_2_N], was examined. Despite the difficulty to detect the subtle anomaly under such large Δ*T*_g_, an analysis based on self-concentration model, which stresses the effective glass transition temperature *T*_g,eff_ of components in binary mixtures, could be performed to reveal this effect. Mok et al. [23] have measured the calorimetric *T*_g,eff_ of PMMA in IL solutions by delicate DSC measurement. Here, we alternatively extract components’ *T*_g,eff_ by the rheological method, the value of is more “dynamic” than “calorimetric”, reflecting the segmental dynamics more properly. Experimental *T*_g_’s from dynamic and calorimetric sources were found to be consistent, but both deviate from the conventional solutions and self-concentration prediction, indicating a retardation of segmental dynamics of PMMA in ILs (see detailed discussion below).

**Figure 2:**
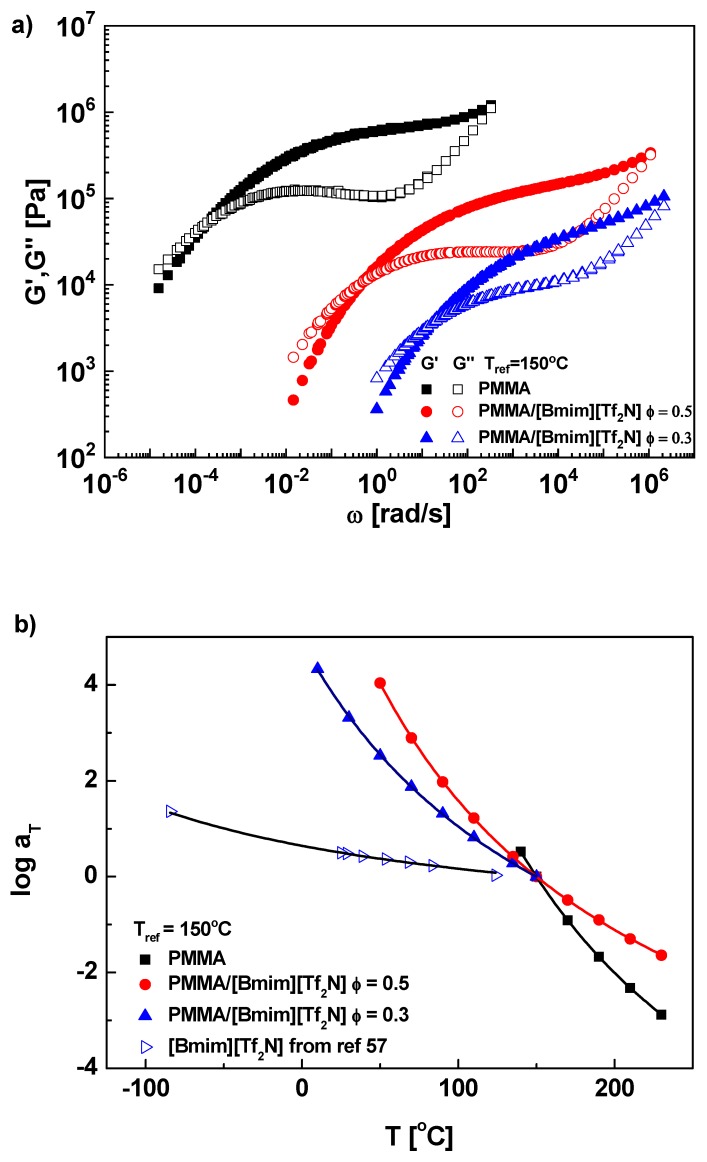

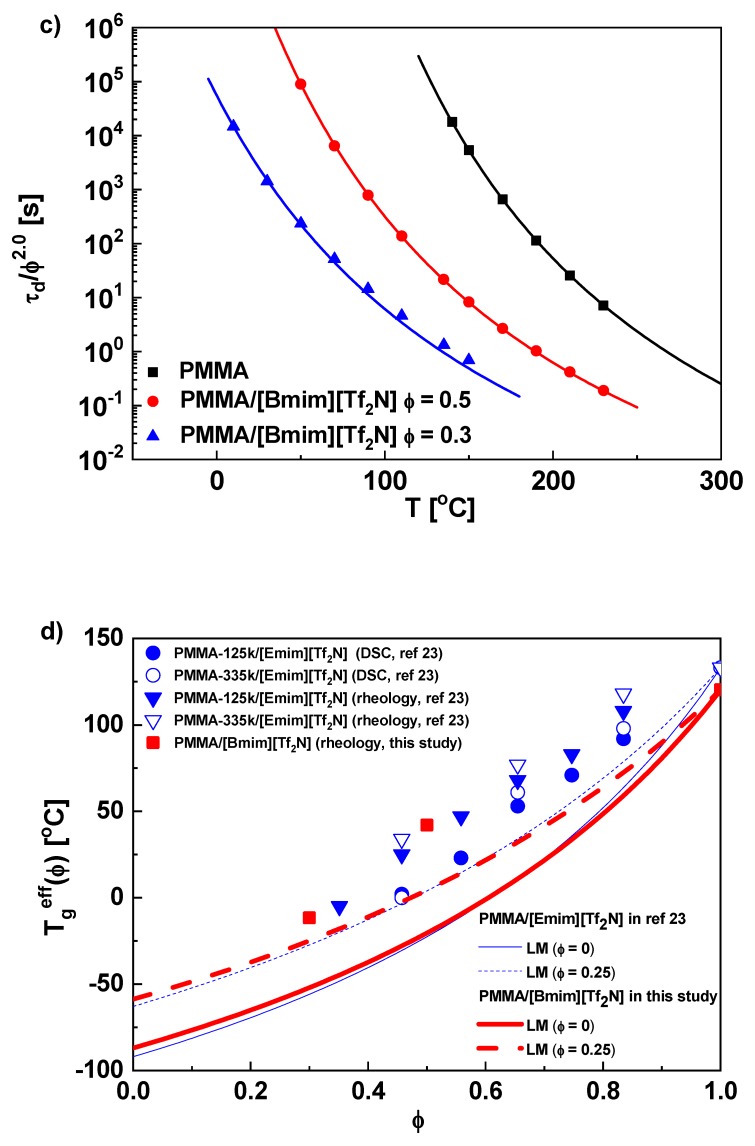

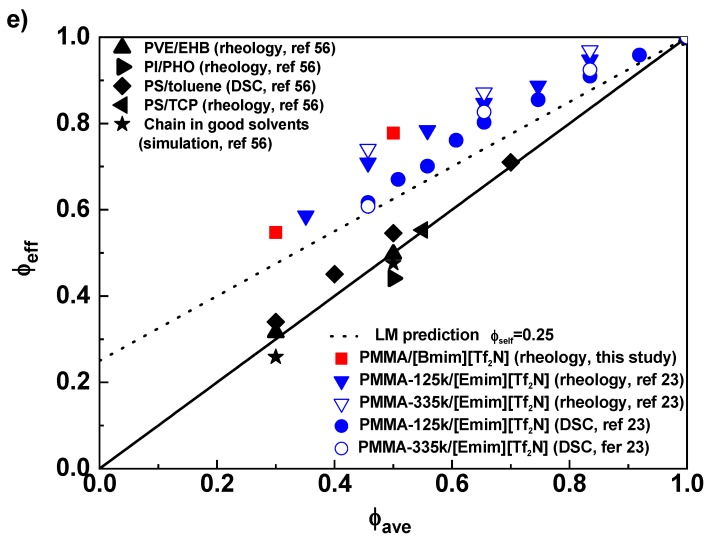
(**a**) The master curve, (**b**) the horizontal shift factor, and (**c**) the normalized terminal relaxation time *τ*_d_/*ϕ*^2.0^ for PMMA and its solutions in [Bmim][Tf_2_N] at *T*_ref_ = 150 °C. (**d**) The effective glass transition temperatures as function of volume fraction *ϕ* for PMMA/[Bmim][Tf_2_N] in this study (■) and PMMA/[Emim][Tf_2_N] in ref [23] (▼,▽ for rheology and ●,○for DSC, unit *k* in legend representing kg/mol). The solid and dotted curves are predictions of LM model with self-concentration *ϕ*_self_ = 0 and 0.25, respectively. (**e**) *ϕ*_eff_ as a function of *ϕ*_ave_ for PMMA in IL solutions. The solid line is the diagonal representing *ϕ*_eff_ = *ϕ*_ave_ (or *ϕ*_self_ = 0), while he dotted curve is prediction of LM model with *ϕ*_self_ = 0.25. Also plotted are data for PVE/EHB (▲), PI/PHO (●), PS/toluene(◆), PS/TCP(◀), and simulated chain in good solvent (★), from ref [56].

Master curves of PMMA/[Bmim][Tf_2_N] solutions are shown in Figure 2a. Due to the strong plasticizing effect (Δ*T*_g_ = 212 °C), the crossover of *G*′ and *G*″, which corresponds to the characteristic relaxation time, significantly shifts to high-frequency with dilution. The respective plateau modulus for *ϕ* = 1, 0.5, and 0.3 samples are 6.3 × 10^5^, 1.3 × 10^5^, and 3.9 × 10^4^ Pa, corresponding to the entanglement number *Z*_exp_ = 99.6, 40.5, and 20.8, so all three samples are sufficiently entangled. The concentration scaling of plateau moduli is *G*_N_^0^~*ϕ*^2.3^, consistent with experimental results for conventional polymer solutions [47], high-*ϕ* (≥0.3) PMMA/[Emim][Tf_2_N] solutions [23], and the Colby-Rubinstein theoretical prediction [45,46]. The smaller exponent than PEO/IL solutions implies that the high-*ϕ* PMMA/IL solutions are more homogeneous and the entanglement is not impaired by interaction with ILs. However, a larger exponent around 2.6 was observed in low-*ϕ* (≤0.2) PMMA/[Emim][Tf_2_N] solutions [23], as the case in low-*ϕ* PEO/ILs. Both PMMA and PEO results seem to indicate the entanglement impairment mainly appears at low polymer concentrations where the polymer-IL interaction overwhelms the polymer-polymer interaction, making the hydrogen-bonding-induced local heterogeneity nonnegligible.

In Figure 2b, the temperature dependence of *a*_T_ monotonically decreases with dilution. The WLF fitting is performed and parameters at *T*_ref_ = 150 °C are *C*_1_ = 10.3 and *C*_2_ = 206 °C for *ϕ* = 1, *C*_1_ = 7.43 and *C*_2_ = 284 °C for *ϕ* = 0.5, *C*_1_ = 5.86 and *C*_2_ = 330 °C for *ϕ* = 0.3, and *C*_1_ = 1.43 and *C*_2_ = 486 °C for *ϕ* = 0, respectively. The corresponding fractional free volumes *f*/*B* are 0.042, 0.058, 0.074, and 0.304, respectively. Apparently, the plasticizing effect in PMMA/ILs is similar with conventional polymer solutions. However, the considerable plasticization due to large Δ*T*_g_ masks the subtle difference in segmental dynamics between IL and conventional solutions. To amplify the difference, we resort to the self-concentration model [73], which can quantitatively evaluate the segmentally dynamic change in binary mixtures. 

The self-concentration model was firstly proposed to interpret the distinct dynamics of two components in miscible polymeric blends [73]. This concept was further applied to polymer solutions, where a weaker self-concentration effect was observed with respect to polymeric blends [56,74,75]. In the self-concentration model, the chain connectivity is stressed so that the effective local concentration *ϕ*_eff_ within cooperative length is estimated to be larger than the average concentration *ϕ*_ave_. Lodge and McLeish (LM) [73] postulate the cooperative length to be Kuhn scale. *ϕ*_eff_ can be written as

(4) $$\phi_{\mathrm{eff}}^{}=\phi_{\mathrm{self}}+(1-\phi_{\mathrm{self}})\phi_{m}^{}$$

with *ϕ*_self_ being the self-concentration, defined as the occupied volume fraction of a polymer chain within cooperative volume, and *ϕ*_m_ being the interchain contribution from identical-species surrounding chains. *ϕ*_m_ is postulated to be *ϕ*_ave_ by LM and evidenced in miscible blends [76]. In solutions, however, *ϕ*_m_ is found smaller than *ϕ*_ave_ [56,74,75]. *ϕ*_eff_ can be obtained from the effective glass transition temperature *T*_g,eff_ according to the Fox equation

(5) $$\frac{1}{T_{g,\mathrm{eff},A}^{}}=\frac{\phi_{\mathrm{eff},A}^{}}{T_{g,A}}+\frac{1-\phi_{\mathrm{eff},A}^{}}{T_{g,B}}$$

with subscripts A and B representing two components. *T*_g,eff_ is the dynamic reference temperature. Experimentally, *T*_g,eff_ can be extracted by fitting the temperature dependence of relaxation times [56,77]. 

In previous work [56], we developed a method to obtain *T*_g,eff_ from the rheological terminal relaxation time in concentrated polymer solutions. First, the terminal relaxation time *τ*_d_ is normalized by a dilution scaling *ϕ^v^*, with exponent *v* being 2.0 [36]. In this way, the contribution of entanglement dilution on the decrease of *τ*_d_ has been eliminated, so one can explicitly investigate the plasticizing effect on segmental dynamics. Then, *τ*_d_/*ϕ*^2^ is plotted as function of temperature and fitted with WLF equation by

(6) $$\log\left(\frac{\tau_{d}/\phi^{2}}{\tau_{d,\mathrm{ref}}}\right)=\frac{-C_{1}\left[T-(T_{\mathrm{ref}}+\Delta T)\right]}{C_{2}+T-(T_{\mathrm{ref}}+\Delta T)}$$

with *τ*_d,ref_, *C*_1_, and *C*_2_ being WLF parameters for pure polymers at reference temperature *T*_ref_. The three parameters have been proved to be independent of composition at an iso-free-volume state [36,56,78,79]. In solutions, the iso-free-volume temperature is *T*_ref_ + Δ*T*, where Δ*T* is determined by horizontally shifting the WLF curve of the pure polymer to superpose the data in solutions. Since polymer solutions at their effective glass transition temperature *T*_g,eff_(*ϕ*) are iso-free-volume with pure polymers at *T*_g_ [41,56,79], the shift factor Δ*T* can be used to calculate *T*_g,eff_(*ϕ*) by [56,79]

(7) $$T_{g,\mathrm{eff}}(\phi )=T_{g}+\Delta T$$

From *T*_g,eff_(*ϕ*), values of *ϕ*_eff_ and *ϕ*_self_ could be calculated according to Equations (5) and (4), respectively.

*τ*_d_/*ϕ*^2^ of PMMA/[Bmim][Tf_2_N] has been shown in Figure 2c, with *τ*_d_ being defined by the reciprocal of *G′–G″* crossover. The WLF fitting curve of pure PMMA is horizontally shifted to fit data of solutions and achieves good overlap, verifying the validity of the “shift” method. *T*_g,eff_(*ϕ*) is extracted from Δ*T* through eq 7. For PMMA/[Bmim][Tf_2_N], *T*_g,eff_(*ϕ*) is higher than the LM prediction (theoretical *ϕ*_self_ = 0.25 for PMMA [73]) (Figure 2d). PMMA/[Emim][Tf_2_N] solutions in ref [23] are also analyzed by our method (*τ*_d_/*ϕ*^2^ vs *T* plots are shown in Figure S3 in Supporting Information). Similarly, *T*_g,eff_(*ϕ*)’s of PMMA in [Emim][Tf_2_N], both from calorimetric and dynamic measurements, are higher than the LM prediction (Figure 2d). The observation of PMMA in ILs conflicts with conventional polymer solutions, whose *T*_g,eff_(*ϕ*) follows the original Fox equation with apparent *ϕ*_self_ ≈ 0 [56,74]. To compare with data from conventional solutions, we plot *ϕ*_eff_ as function of *ϕ*_ave_ in Figure 2e. For PMMA in ILs, *ϕ*_eff_ is universally higher than the LM prediction, while for conventional polystyrene (PS), 1,2-polybutadiene (PVE), 1,4-polyisoprene (PI), and simulated solutions, *ϕ*_eff_ is approximately equal to *ϕ*_ave_ [56]. The smaller *ϕ*_eff_ in conventional solutions has been ascribed to the smaller *ϕ*_m_ than *ϕ*_ave_ [56], while the large *ϕ*_eff_ and *T*_g,eff_(*ϕ*) in IL solutions should be attributed to a retarded segmental dynamics due to polymer-IL interactions. Indeed, as the case in PEO/IL solutions, the carbonyl oxygens on PMMA monomers may coordinate with cations on ILs to impede the segmental motion. This effect makes experimentally obtained *T*_g,eff_ (and hence *ϕ*_eff_) apparently larger, although the local concentration might not be effectively changed by the interaction.

The polymer-IL interaction could retard not only dynamics of polymers, but also solvents. For example, Mok et al. [23] observed that *T*_g_ of [Emim][Tf_2_N] increases as far as 50 °C when it is blended with PMMA at a concentration of 50%. For a conventional solution with a comparable Δ*T*_g_ and concentration, for example, polystyrene (PS)/ di-n-butyl phthalate (DBP), the increase in *T*_g_ of solvent is only about 25 °C [74]. This result supports the existence of polymer-IL interaction from the solvent aspect.

## 4. Nonlinear Viscoelasticity

### 4.1. Start-up Measurement

**Figure 3:**
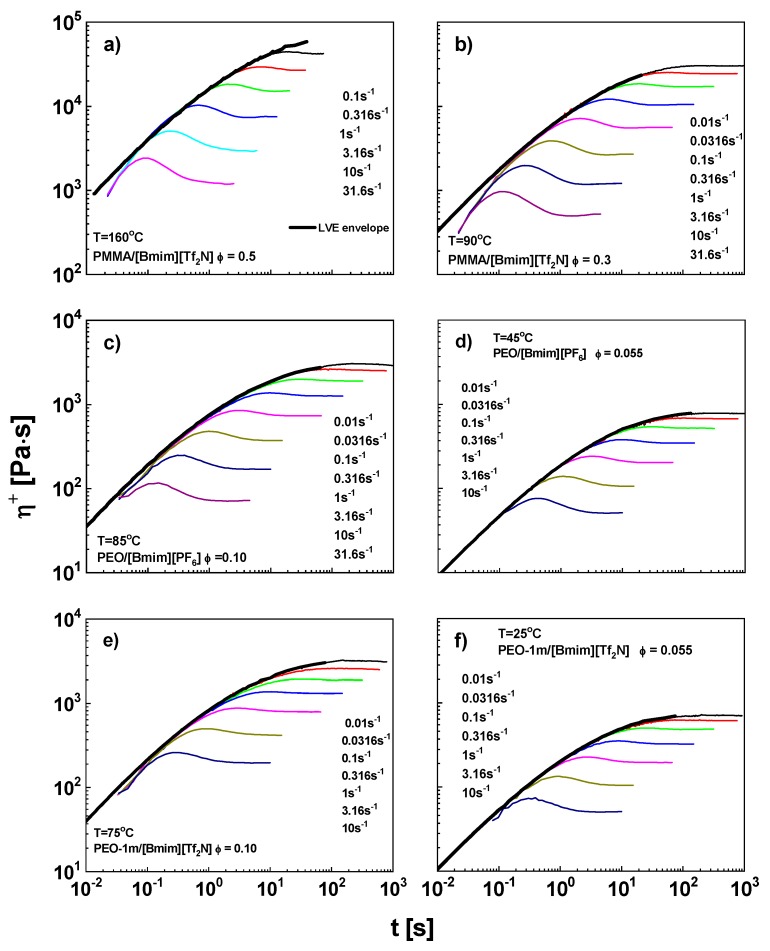
Nonlinear startup shear viscosity *η*^+^ (thin curves) as a function of time for PMMA/[Bmim][Tf_2_N] with *ϕ* = (**a**) 0.50 and (**b**) 0.30, for PEO/[Bmim][PF_6_] with *ϕ* = (**c**) 0.10 and (**d**) 0.055, and for PEO/[Bmim][Tf_2_N] with *ϕ* = (**e**) 0.10 and (**f**) 0.055. In each panel, the shear rates increase from top to bottom with a logarithmic interval of 0.5. The thick curves are LVE envelopes.

The transient viscosity *η*^+^ for PEO/ILs and PMMA/ILs is shown in Figure 3. The LVE envelopes are converted from master curves according to the empirical Cox-Merz rule [80], $\eta \left(\overset{\cdot }{\gamma }\right)={\eta^{*}\left(\omega \right)|}_{\omega =\overset{\cdot }{\gamma }}$, in conjunction with the Gleissle rule [81], $\eta^{+}\left(t\right)={\eta \left(\overset{\cdot }{\gamma }\right)|}_{\overset{\cdot }{\gamma }=1/t}$, with η^*^ being the complex viscosity, $\overset{\cdot }{\gamma }$ being shear rate, and *ω* being frequency. The *η*^+^ data below 0.1 s deviate from the envelope due to the start-up artefacts of motor. Beyond 0.1 s, the *η*^+^ curve matches the LVE envelope. At longer time, *η*^+^ departs from envelopes again and exhibits an overshoot, after which, *η*^+^ reaches its steady value *η*_steady_. With the increasing shear rate, the overshoot appears at shorter time and visually becomes prominent. These features are consistent with those in linear polymer melts and solutions [33,82,83,84,85]. 

**Figure 4:**
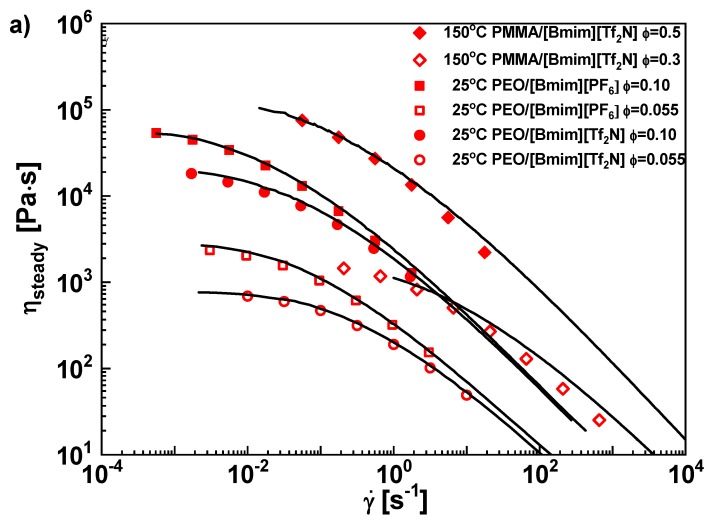

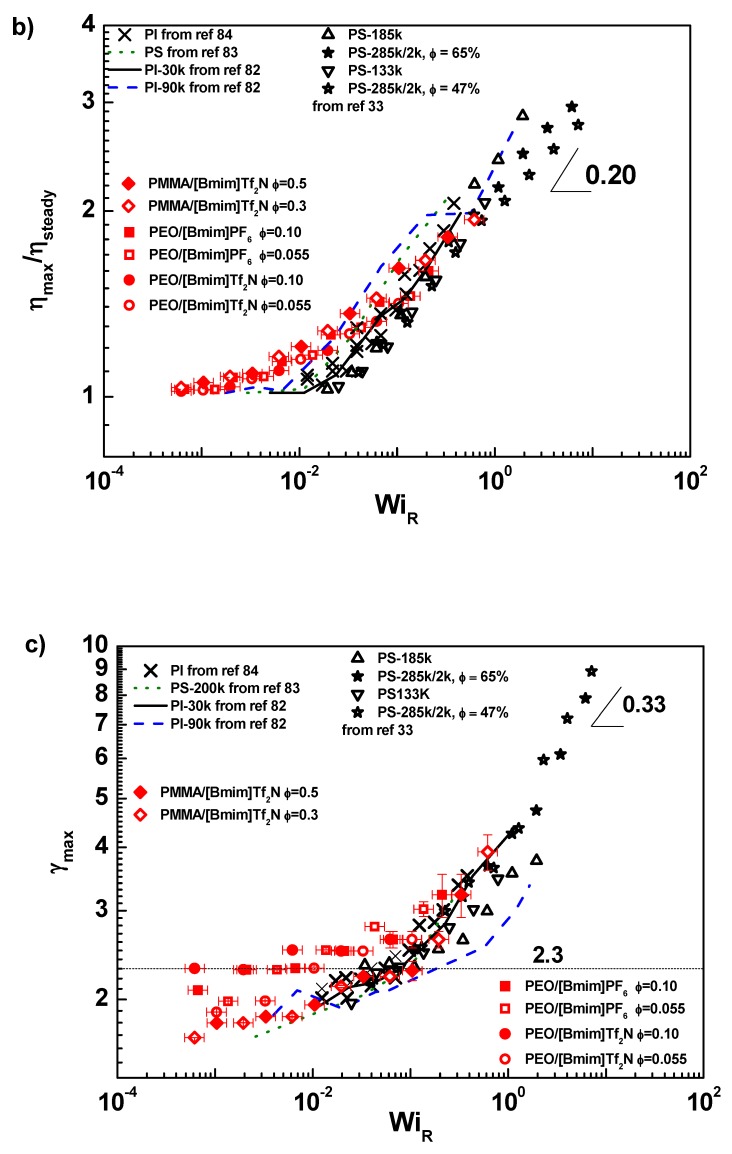
(**a**) Steady shear viscosities of PEO/IL solutions as a function of shear rate. The solid curves are from LVE results. (**b**) The maximum viscosity divided by steady viscosity *η*_max_/*η*_steady_, as a function of Rouse Weissenberg number *Wi*_R_. (**c**) The strain where the maximum transient viscosity appears, *γ*_max_, as a function of *Wi*_R_. The legend for all panels: (■) PEO/[Bmim][PF_6_], *ϕ* = 0.10, (●) PEO/[Bmim][Tf_2_N], *ϕ* = 0.10, (□) PEO/[Bmim][PF_6_], *ϕ* = 0.055, (○) PEO/[Bmim][Tf_2_N], *ϕ* = 0.055, (◆) PMMA/[Bmim][Tf_2_N], *ϕ* = 0.50, (◇) PMMA/[Bmim][Tf_2_N], *ϕ* = 0.30, (△) PS-185 kg/mol (ref [33]), (▽) PS-133 kg/mol (ref [33]), (★) PS-285 kg/mol in PS-2 kg/mol solution, *ϕ* = 65% (ref [33]), (☆) PS-285 kg/mol in PS-2 kg/mol solution, ϕ= 47% (ref [33]), (∙∙∙∙∙) PS-200 kg/mol (ref [83]), (—) PI-30 kg/mol (ref [82]), (-----)PI-90 kg/mol (ref [82]), (×) PI (ref [84]).

The steady viscosity *η*_steady_ is extracted by averaging over the steady portion on the start-up curve. The standard deviation is negligible and covered by the size of symbols. *η*_steady_ is plotted as function of shear rate in Figure 4a. Also plotted are the LVE envelopes transformed from the complex viscosity η^*^(*ω*). Shear thinning is observed in all samples. *η*_steady_ overlaps with the LVE envelopes well, confirming the validity of the Cox-Merz rule [80]. We note that in cellulose/IL solutions [86,87,88,89], the Cox-Merz rule was found to fail, where steady viscosity exhibits a weaker shear thinning behavior than complex viscosity. This unusual phenomenon was tentatively explained by the increased possibility of temporary intermolecular association when shear flow expands coils to improve their contacts [86]. However, since both PEO and PMMA are polymers without mutual hydrogen bonding association, the Cox-Merz rule still works here. 

Figure 4b plots the relative viscosity, *η*_max_/*η*_steady_, as function of the Rouse Weissenberg number *Wi*_R_. *η*_max_/*η*_steady_ is a measure proportional to the maximum deformation at steady state, where *η*_max_ is the maximum transient viscosity obtained through a Gaussian fit of the peak. The uncertainty of *η*_max_/*η*_steady_ is covered by symbols. *Wi*_R_ is calculated by *τ*_Rouse_ $\overset{\cdot }{\gamma }$ with *τ*_Rouse_ being the Rouse relaxation time defined by *τ*_d_/3*Z*_est_ [84,90]. The error of *Wi*_R_ are from determination of *τ*_d_, about 0.1 decade on the frequency axis. The *η*_max_/*η*_steady_ data are independent of shear rate and slightly larger than 1 when *Wi*_R_ < 0.01. Above 0.01, *η*_max_/*η*_steady_ starts to monotonically increase and approach the scaling exponent of 0.2, which was observed in linear polymer melts [84]. Our data fail to exceed the chain-stretching shear rate (*Wi*_R_ > 1) before sample fracture, meaning that most chains are orientation-dominating under flow. However, we note that our samples are slightly polydisperse, but the selected *τ*_d_ is more reflecting the low-*M*_w_ side. Therefore, even though *Wi*_R_ is lower than 1, some high-*M*_w_ fraction might have already experienced stretching. Interestingly, data of polymer/ILs overlap with literature results of pure PS [83], pure PI [82,84], and PS/oligomer-styrene solutions [33], reflecting the universality of chain deformation under shear flow in different environments. Indeed, as found by rheological [16] and small-angle neutron scattering [17] measurements, some imidazolium ILs, e.g., [Bmim][PF_6_] and [Bmim][Tf_2_N] as used in this research, are either θ or moderately good solvents for PEO, so conformations of coils in these ILs are similar to those in melts and conventional solvents. Moreover, the minimum time scale of nonlinear response is usually as large as 0.01 s, greatly exceeding the time scale for dissociation and exchange of the coordinating ion (in the order of tens of nanoseconds). Therefore, the nonlinear response of polymer chains cannot sense the confinement from polymer-IL interaction, although the interaction is crucial for the segmental dynamics.

Figure 4c shows the strain at the maximum viscosity, *γ*_max_, as function of *Wi*_R_. *γ*_max_ denotes the maximum instantaneous chain deformation under flow, as predicted by tube model-based theories [91,92] and partially supported by small-angle neutron scattering experiment [93,94]. The uncertainty of *γ*_max_ is estimated as the product of shear rate and the time resolution of data acquisition (~0.01s). The *γ*_max_ values of IL solutions locate between 2.0 and 2.3 when *Wi*_R_ < 0.1. This result is consistent with reported experiments [84] and the Doi-Edwards prediction [82,83,91,95], indicating that the tube orientation dominates the overshoot at low shear rate. When *Wi*_R_ > 0.1, *γ*_max_ starts to increase with *Wi*_R_ following a scaling slope of 0.33, implying that both chain orientation and stretch contribute to the overshoot (although the orientation is still dominating). The slope 0.33 is lower than the slope of 1 predicted by GLaMM theory [92], but could be tentatively rationalized by invoking a minimum internal force required for the chain retraction [33,82]. Interestingly, *γ*_max_ of IL solutions again overlaps with the literature results from pure PS [83], pure PI [82,84], and PS/oligomer styrene solutions [33]. Thus, it is speculated that the shear-induced chain deformation in ILs share the same mechanism as in other environments. 

Two parameters that govern the rheology of entangled polymers are the entanglement number *Z* and the monomeric friction coefficient *ζ*. Polymer melts and solutions have the same linear viscoelastic spectrum in dimensionless coordinates if they have the same *Z* [33]. However, understanding the nonlinear rheological behavior in melt and solution is more complicated. In nonlinear extensional rheology, the steady-state extensional viscosity of polymer melts exhibits a stretch rate thinning behavior, while for polymer solutions with the same *Z*, the steady-state extensional viscosity gradually changes to stretch rate thickening by decreasing polymer concentrations [31,96]. This difference can be ascribed to the chain-alignment-induced reduction of monomeric friction *ζ*, whose effect is more significant in polymer melts but attenuated in solutions due to the average of the strongly-aligned chain segments and the less-aligned solvent molecules [31,34,35]. Furthermore, the anisotropic friction in solutions leads to polymer-solvent nematic interactions, making the chain alignment dependent on solvent species [32,34]. In nonlinear start-up shear rheology, the difference between the melt and solutions with the same *Z* has disappeared because a chain tumbling happens under shear flow, which substantially reduces the monomeric alignment [33]. Indeed, our polymer/IL solutions exhibits a universal start-up shear response with other melts and solutions, which represents the explanation of chain tumbling also account for our results. Considering the limited research in extensional rheology of IL solutions [97], a challenging topic in future is to impose systematic extensional measurement on entangled polymer/IL solution to investigate the difference among melts, conventional solutions, and IL solution, especially the orientational coupling parameter *ϵ*, which measures the polymer-solvent nematic interaction and depends on solvents. In addition, neutral polymers used in this study do not have significant interactions with each other and solvents. However, strong hydrogen bond [86] and electrostatic [98] interaction were found to effectively change the concentration scaling of viscosity in IL solutions. The influence of these interactions on start-up rheology is to be clarified in future studies by using hydrogen-bond polymers and polyelectrolyte.

### 4.2. Nonlinear Stress Relaxation

**Figure 5:**
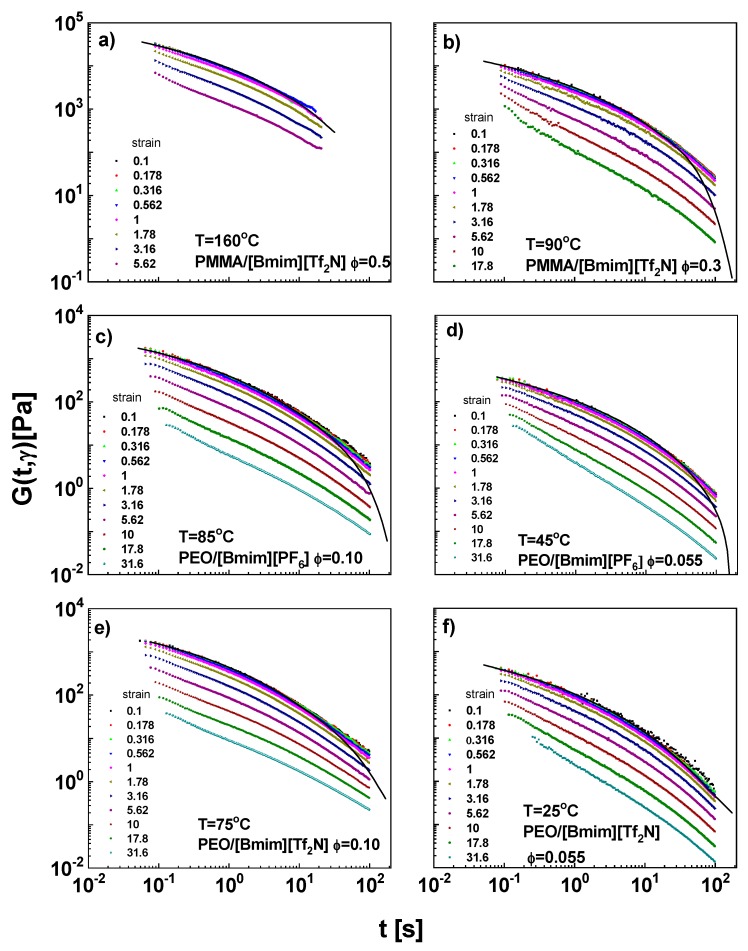
The stress relaxation moduli for (**a**) PMMA/[Bmim][Tf_2_N], *ϕ* = 0.5, 160 °C, (**b**) PMMA/[Bmim][Tf_2_N], *ϕ* = 0.3, 90 °C, (c) PEO/[Bmim][PF_6_], *ϕ* = 0.10, 85 °C, (d) PEO/[Bmim][PF_6_], *ϕ* = 0.055, 45 °C, (e) PEO/[Bmim][Tf_2_N], *ϕ* = 0.10, 75 °C, and (f) PEO/[Bmim][Tf_2_N], *ϕ* = 0.055, 25 °C. The solid curves are the *G*(*t*,*γ*) when γ→0, converted from master curves.

The stress relaxation moduli *G*(*t*, *γ*) after different imposed step strains were measured (Figure 5). For several small strains, *G*(*t*, *γ*) curves collapse on the LVE envelopes (converted from complex moduli by modeling with discrete spectrum). At larger strains, *G*(*t*, *γ*) curves drop progressively with increasing strain, corresponding to nonlinear viscoelasticity. *G*(*t*, *γ*) can be factorized as the product of two independent components, the time-dependent modulus *G*(*t*) in the linear regime of deformation and the strain-dependent damping function, *h*(*γ*):

(8) G(t,γ)=G(t)h(γ)

**Figure 6:**
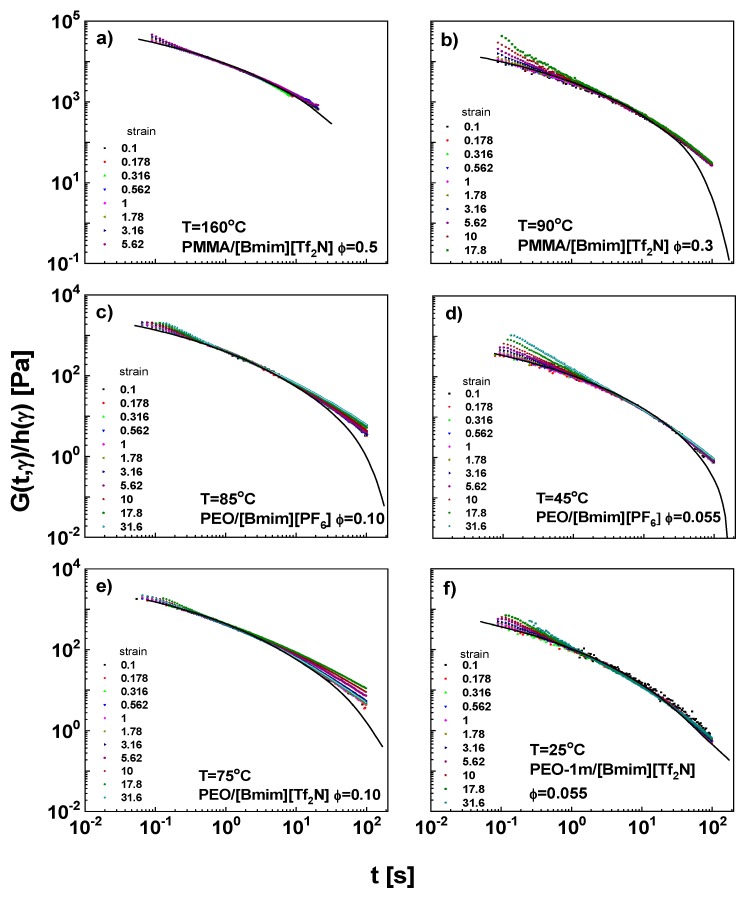
The stress relaxation moduli after vertical shifting for (**a**) PMMA/[Bmim][Tf_2_N], *ϕ* = 0.5, 160 °C, (**b**) PMMA/[Bmim][Tf_2_N], *ϕ* = 0.3, 90 °C, (**c**) PEO/[Bmim][PF_6_], *ϕ* = 0.10, 85 °C, (**d**) PEO/[Bmim][PF_6_], *ϕ* = 0.055, 45 °C, (**e**) PEO/[Bmim][Tf_2_N], *ϕ* = 0.10, 75 °C, and (**f**) PEO/[Bmim][Tf_2_N], *ϕ* = 0.055, 25 °C. Symbols are the same with Figure 5.

The damping function *h*(*γ*) is a measure of remaining stress to be relaxed after rapid chain retraction [99], reflecting conformational change of chains under large strain. It can be determined by vertically shifting *G*(*t*, *γ*) curves at large strains to the LVE envelopes. After the shift (Figure 6), overlap is achieved, suggesting a successful time-strain separability for these IL solutions. The overlap failure at short-*t* limits stems from the start-up artefact of the motor, while the failure at high-*t* limits is due to the torque resolutions, which suffers a poor signal-to-noise ratio at low torque.

**Figure 7:**
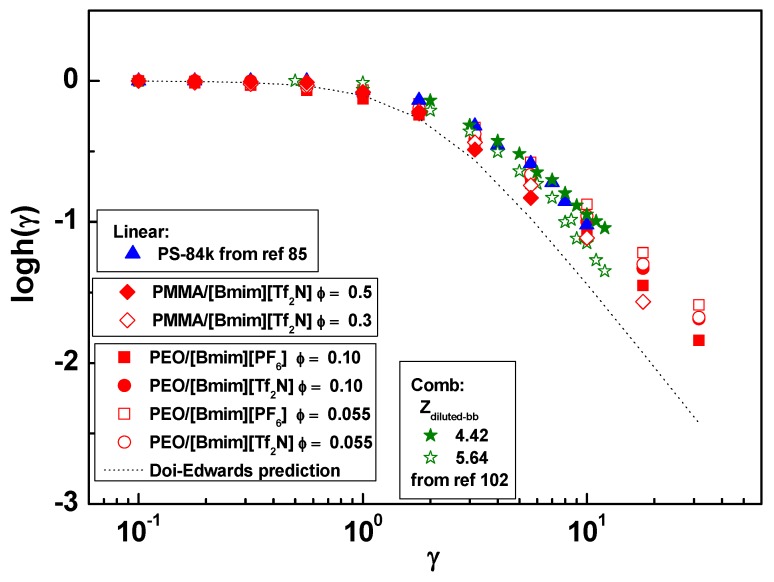
Damping functions of (■) PEO/[Bmim][PF_6_], *ϕ* = 0.10, (●) PEO/[Bmim][Tf_2_N], *ϕ* = 0.10, (□) PEO/[Bmim][PF_6_], *ϕ* = 0.055, (○) PEO/[Bmim][Tf_2_N], *ϕ* = 0.055, (◆) PMMA/[Bmim][Tf_2_N], *ϕ* = 0.50, (◇) PMMA/[Bmim][Tf_2_N], *ϕ* = 0.30, (▲) linear PS—84 kg/mol (ref [85]), (★) comb PS with diluted backbone entanglement number *Z*_diluted-bb_ = 4.42 (ref [102]), and (☆) comb PS with diluted backbone entanglement number *Z*_diluted-bb_ = 5.64 (ref [102]). The dotted curve is the Doi-Edwards prediction.

The extracted *h*(*γ*) is plotted in Figure 7. The *h*(*γ*) values are strain-independent when *γ* < 1 and then decrease with increasing *γ*. A close inspection reveals that the strain thinning becomes slightly significant with increasing entanglement number *Z*. This tendency is consistent with the observation in linear polymer melts, where the highly entangled polymers show strain thinning close to the Doi-Edwards (DE) prediction [100], while the less entangled polymers deviate towards Rouse behavior (*h* (*γ*) = 1) [100,101]. Also plotted are *h*(*γ*) from linear PS melt [85] and dynamically diluted backbone on comb PS melt [102]. They are marginally entangled and overlap well with our less-entangled PEO/IL solutions. Experimental data are compared with the Doi-Edwards (DE) prediction without independent alignment approximation [102], $h(\gamma )=\frac{1}{1+\frac{4}{15}\gamma^{2}}$, which was developed for highly entangled linear chains. The weaker strain dependence of experimental *h*(*γ*) indicates the moderate entanglement of investigated samples cannot match the ideally high entanglement in DE model. Another reason for the deviation might be from the slightly broad polydispersity. As shown in literature [103,104,105], broad polydispersity results in a less pronounced dependence on strain because the long chain portions are only partially retracted, while the short chain portions reptate. The average response is reflected as the weaker strain thinning than the DE prediction. However, we note that the polydispersity of our polymers is not as broad as those in literatures [103,104,105], which are mostly higher than 2.0. Therefore, the contribution of polydispersity should be relatively small.

Before this study, it is an unsolved problem that whether the rapid chain retraction after step strain could be affected by the polymer-IL interactions. Our experiments reveal that the hydrogen bonding interaction between oxygen and cations cannot significantly change the chain recovery after large deformation, similar with the case under shear flow. As a result, the damping functions of polymer/IL solutions are consistent with the literature results from melts. In future studies, stronger polymer-IL interaction is desired to be constructed to amplify their effect on nonlinear stress relaxation. To this end, polyelectrolytes/IL solutions, ionomer/IL solutions, and strong hydrogen-bonding pairs (e.g., cellulose/1-ethyl-3-methylimidazolium methylphosphonate [86]) are possible candidates. We hope that the present data will motivate more experimental and modeling/simulation works in this direction.

## 5. Conclusions

An anomalous plasticizing effect has been found in polymer/ionic liquid (IL) solutions. PEO/IL solutions, which have small Δ*T*_g_, exhibit stronger temperature dependence of *a*_T_ than pure PEO, in conflict with the normal plasticizing effect that lower-*T*_g_ solvent will increase the fractional free volume. For PMMA/IL solutions with large Δ*T*_g_, *T*_g,eff_ of PMMA in solutions is higher than the prediction by Lodge-McLeish self-concentration model and in contradiction to the case in conventional polymer solutions, where *T*_g,eff_ of polymers obeys the predictions using the original Fox equation. Both observations reveal the fact that the segmental dynamics of neutral polymers are retarded in ILs compared with conventional polymer solvents. This argument is supported by a recent simulation result which demonstrates that polymer segments wrap the cations of ILs due to oxygen-cation interactions, and the unwrapping of segments delays the segmental relaxation. Start-up shear and nonlinear stress relaxation measurements indicate a universal nonlinear rheological behavior for polymer/IL solutions as linear polymer melts and solutions. These results imply that the segment-cation interaction is still too weak to influence the nonlinear chain orientation/stretch under shear flow or large deformation, even though it plays an important role in segmental dynamics. The present data are expected to motivate the experimental study on the dynamics of polymer/IL solutions with stronger interactions, which might result in more retardation on the segmental dynamics and make the nonlinear response unusual. Theoretical and simulation developments in this field are also highly desired.

## Supplementary Materials

The following are available online at https://www.mdpi.com/2073-4360/11/5/877/s1. Figures S1 and S2: DSC traces of all investigated samples, Figure S3: The temperature dependence of *τ*_d_/*ϕ*^2^ for PMMA/[Emim][Tf_2_N] solutions.
