# Supplementary material for: Rheology of Concentrated Polymer/Ionic Liquid Solutions: An Anomalous Plasticizing Effect and a Universality in Nonlinear Shear Rheology
Source: Polymers (Basel). 2019 May 14;11(5):877. doi: 10.3390/polym11050877 (PMC6572180; doi:10.3390/polym11050877)
Supplement: Supplementary file 1 [file polymers-11-00877-s001.pdf]

Supporting Information

Rheology of concentrated polymer/ionic liquid solutions: an anomalous plasticizing effect and a universality in nonlinear shear rheology

Zhonghua Liu,[a] Wei Wang,[a] Florian J. Stadler,[a] Zhi-Chao Yan [*, a]

[a] College of Materials Science and Engineering, Shenzhen Key Laboratory of Polymer Science and Technology, Guangdong Research Center for Interfacial Engineering of Functional Materials, Nanshan District Key Lab for Biopolymers and Safety Evaluation, Shenzhen University, Shenzhen 518055, P. R. China.

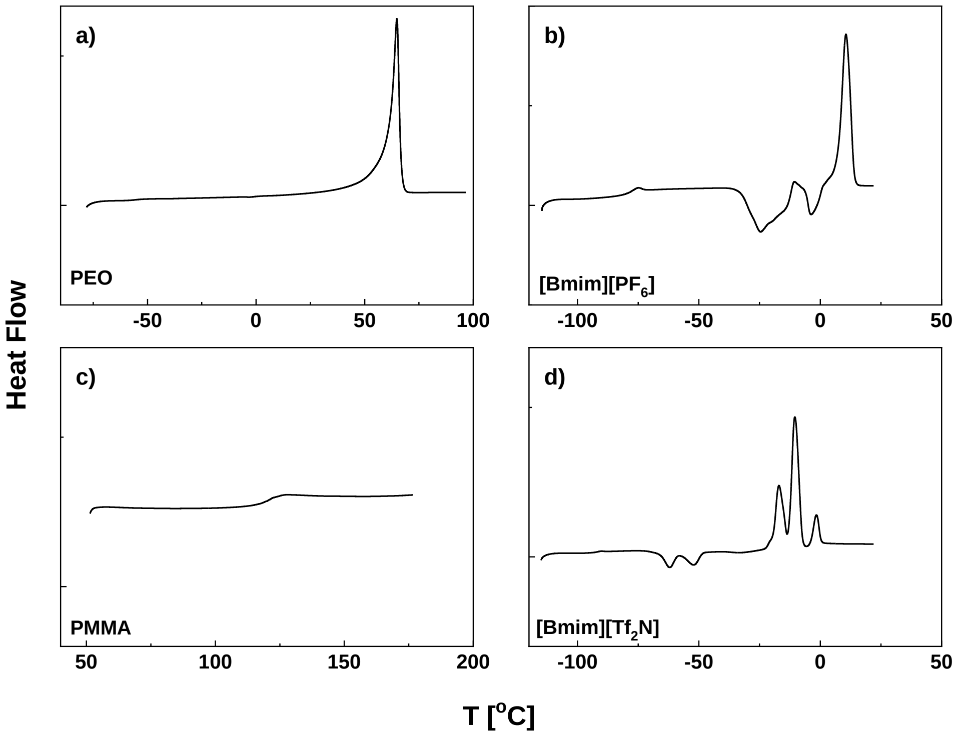

Figure S1. DSC traces for (a) PEO, (b) $[Bmim][PF_6]$, (c) PMMA, and (d) $[Bmim][Tf_2N]$ obtained at 10 °C/min heating. Endothermal heat flow is up.

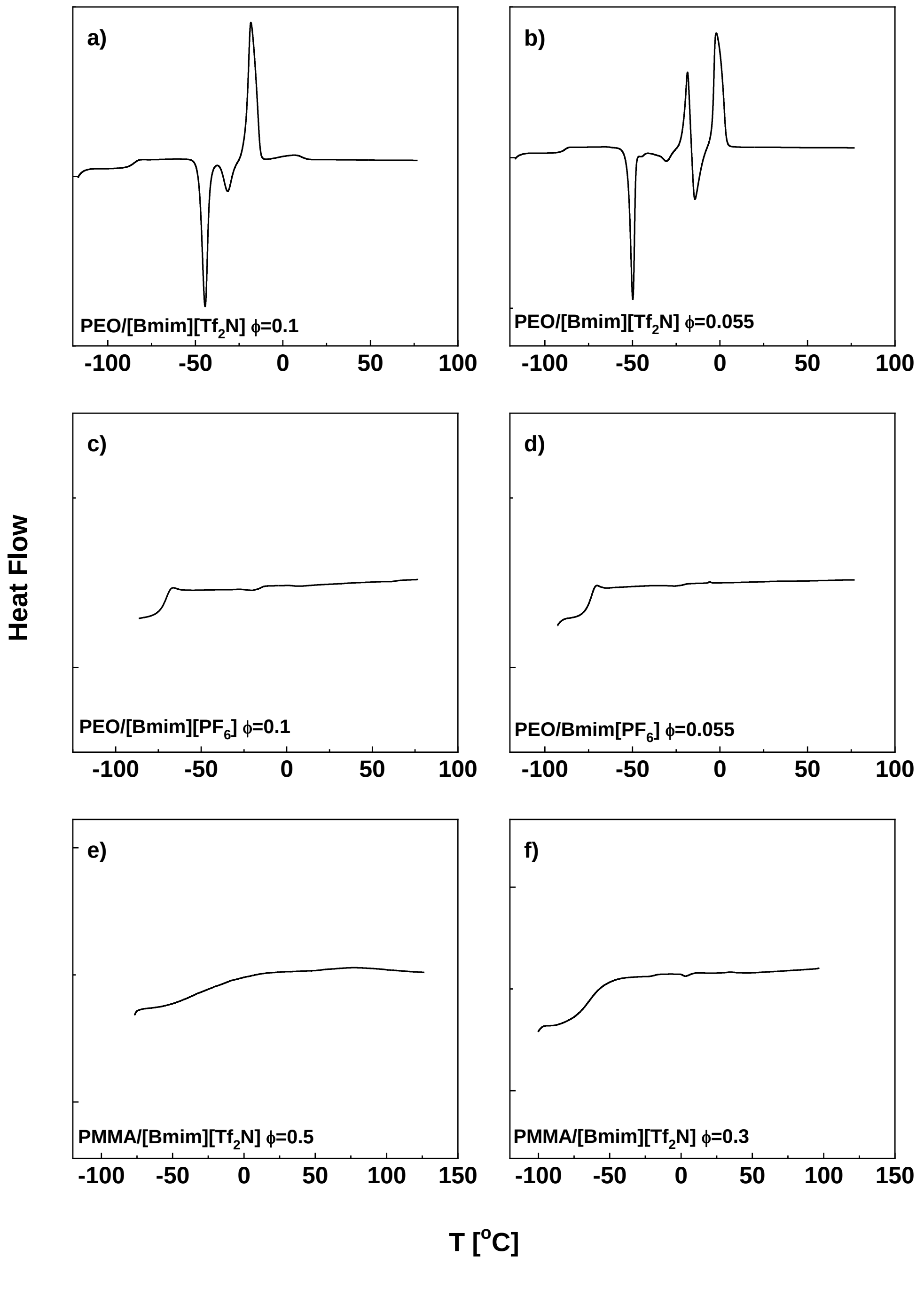

Figure S2. DSC traces for polymer/IL solutions obtained at 10 °C/min heating. Endothermal heat flow is up.

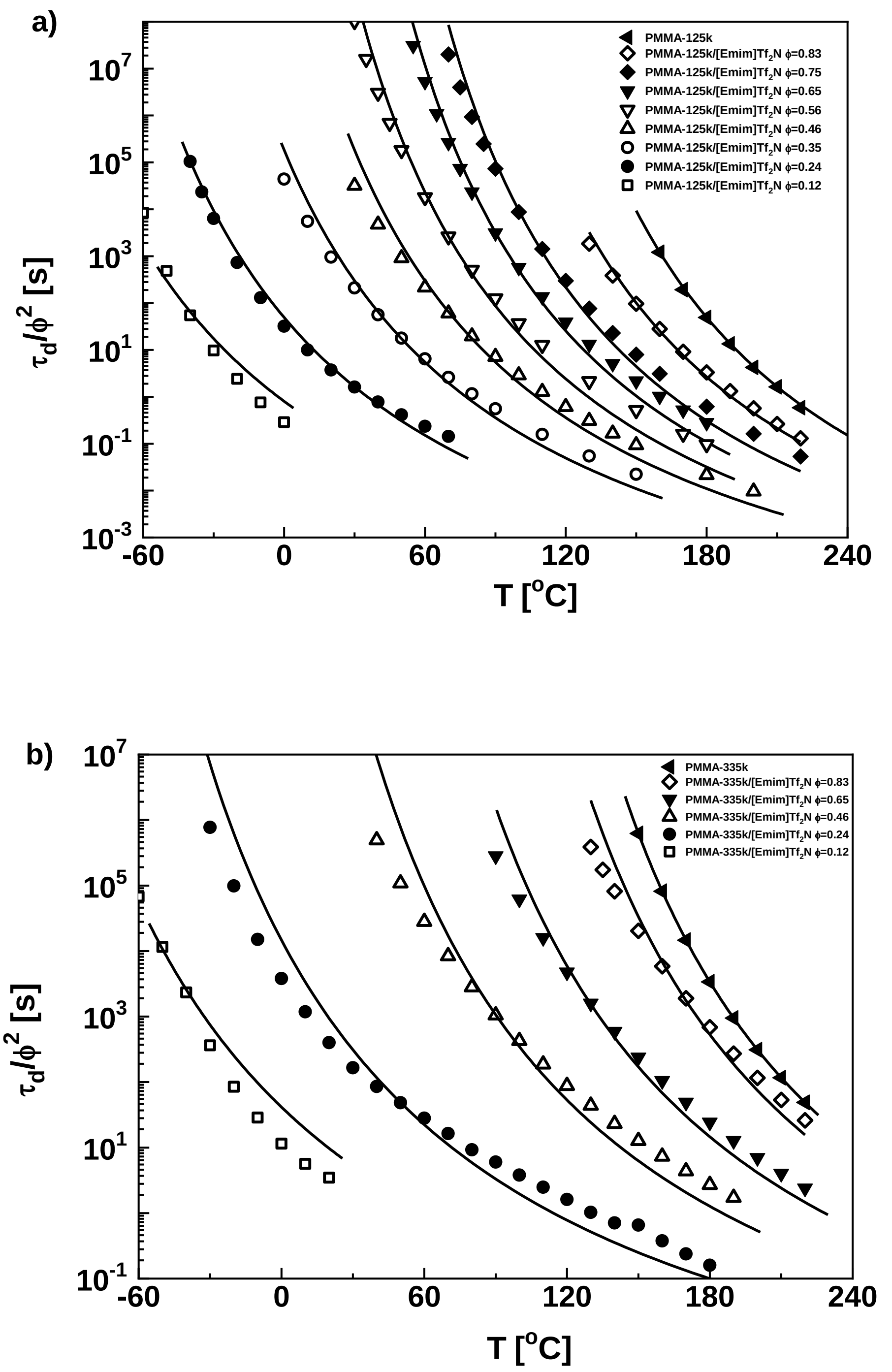

Figure S3. The normalized terminal relaxation time $\tau_d/\phi^{2.0}$ for (a) PMMA-125kg/mol and (b) PMMA-335kg/mol and their solutions in 1-ethyl-3-methaylimidazolium bis(trifluoromethanesulfonyl)imide ([Emim][Tf_2N]). The curves are horizontal shift of WLF fitting for pure PMMA. Data are from ref [1].

Reference

1. Mok, M.M.; Liu, X.C.; Bai, Z.F.; Lei, Y.; Lodge, T.P. Effect of concentration on the glass

transition and viscoelastic properties of poly(methyl methacrylate)/ionic liquid solutions. *Macromolecules* **2011**, *44*, 1016-1025.
